# Gut Microbial Diversity and Community Structure Are Largely Similar Between Apparently Healthy Elderly Japanese Males and Females: A Shotgun Metagenomic Study

**DOI:** 10.3390/life16020297

**Published:** 2026-02-09

**Authors:** M. H. Mahbub, Ryosuke Hase, Natsu Yamaguchi, Yoshiyuki Asai, Masanori Harada, Naoyuki Ichimura, Yoshihiro Hayakawa, Yusuke Inohana, Yutaka Umakoshi, Ryo Yamaguchi, Ren Kimura, Hisashi Tsujimura, Mitsuharu Matsumoto, Fumiaki Higashijima, Takuya Yoshimoto, Kazuhiro Kimura, Tsunahiko Hirano, Keiji Ohishi, Keiko Doi, Kazuto Matsunaga, Tsuyoshi Tanabe

**Affiliations:** 1 Department of Public Health and Preventive Medicine, Graduate School of Medicine, Yamaguchi University, Ube 755-8505, Japan; hossain@yamaguchi-u.ac.jp (M.H.M.); hase@yamaguchi-u.ac.jp (R.H.); natsu@yamaguchi-u.ac.jp (N.Y.); masa.harada@mac.com (M.H.); ichimura.naoyuki.02@pref.yamaguchi.lg.jp (N.I.); 2 Division of Systems Medicine and Informatics, Research Institute for Cell Design Medical Science, Yamaguchi University, Ube 755-8505, Japan; asai@yamaguchi-u.ac.jp; 3 Department of Systems Bioinformatics, Graduate School of Medicine, Yamaguchi University, Ube 755-8505, Japan; 4 AI Systems Medicine Research and Training Center, Graduate School of Medicine, Yamaguchi University Hospital, Yamaguchi University, Ube 755-8505, Japan; 5 Support Center for Rural Medicine, Yamaguchi Prefectural Grand Medical Center, Hofu 747-8511, Japan; 6 Shimadzu Corporation, Kyoto 604-8511, Japan; yo-haya@shimadzu.co.jp (Y.H.); ino-yu@shimadzu.co.jp (Y.I.); umakoshi.yutaka.xm4@shimadzu.co.jp (Y.U.); ryama@shimadzu.co.jp (R.Y.); 7 Analytical Science Research, Kao Corporation, Sumida-ku, Tokyo 131-8501, Japan; kimura.ren@kao.com (R.K.); tsujimura.hisashi@kao.com (H.T.); 8 Research Laboratories, Kyodo Milk Industry Co., Ltd., Tokyo 190-0182, Japan; m-matumoto@meito.co.jp; 9 Department of Ophthalmology, Yamaguchi University School of Medicine, Ube 755-8505, Japan; higashi@yamaguchi-u.ac.jp (F.H.); takuya-y@yamaguchi-u.ac.jp (T.Y.); k.kimura@yamaguchi-u.ac.jp (K.K.); 10 Department of Respiratory Medicine and Infectious Disease, Graduate School of Medicine, Yamaguchi University, Ube 755-8505, Japan; tsuna@yamaguchi-u.ac.jp (T.H.); ohishk@yamaguchi-u.ac.jp (K.O.); decem119@yamaguchi-u.ac.jp (K.D.);; 11 Department of Pulmonology and Gerontology, Graduate School of Medicine, Yamaguchi University, Ube 755-8505, Japan

**Keywords:** gut microbiota, shotgun metagenomics, sex differences, elderly, microbial diversity

## Abstract

Sex differences in gut microbiota may affect health and aging, but evidence in elderly populations is limited and inconsistent. This study examined sex-specific similarities and differences in gut microbiota diversity and composition among apparently healthy elderly Japanese individuals using shotgun metagenomic sequencing. A cross-sectional study was conducted in 100 community-dwelling adults aged 75–83 years (54 males, 46 females). Fecal samples underwent metagenomic sequencing. Alpha and beta diversity were assessed across six taxonomic levels, and taxonomic differences were evaluated using non-parametric tests. No significant sex differences were observed in alpha diversity indices (Shannon, Simpson, evenness, Chao1) at any taxonomic level. Beta diversity based on Bray–Curtis dissimilarity and PCoA also showed no sex-specific clustering. However, certain taxa differed in relative abundance. Males showed higher abundances of *Bacteroidota* (phylum), *Bacteroidia* and *Betaproteobacteria* (class), and *Bacteroidales* and *Burkholderiales* (order) (*p* < 0.05). No significant differences were detected at the family, genus, or species levels. Overall, gut microbial diversity and community structure were largely similar between elderly males and females, with only modest sex-associated differences at higher taxonomic levels. These findings suggest that biological sex may have a limited influence on gut microbiota composition in advanced age and provide population-level reference data for future longitudinal and interventional studies in elderly cohorts.

## 1. Introduction

The world’s population is rapidly aging, leading to a higher prevalence of various diseases and associated disabilities among the elderly. With aging, the gut microbiota undergoes age-related changes [1], often characterized by reduced microbial diversity [2]. In humans, the gut microbiota plays a crucial role in host homeostasis and health, by continuously contributing to metabolism, digestion, nutrition, and host immune regulation [3].

Numerous studies have demonstrated that alterations in the gut microbiota, or dysbiosis, may trigger various pathogenic processes, potentially resulting in a wide range of health disorders, including endocrine and metabolic diseases, immune-mediated conditions, cardiovascular and neurologic disorders, and various cancers [4,5,6]. Accordingly, studies suggest that targeted modulation of the gut microbiota could be a potential strategy to prevent or manage diseases and may promote healthy aging [7,8]. Therefore, a clearer understanding of the baseline gut microbiota composition in healthy elderly is essential, as this knowledge could inform microbiota-modulating strategies for maintaining a healthy gut microbiome and promoting healthy aging.

In the existing literature, biological sex has been recognized as a key determinant of disease susceptibility and pathogenesis, as well as disease prevention, progression, and prognosis of various adverse health outcomes [9,10]. Sex has also been associated with gut microbiome composition and circulating metabolites [11,12]. Therefore, to develop tailored microbiota-modulating interventions for the elderly, a clear understanding of their sex-specific composition is essential. Also, an important question is whether the same intervention would be applicable to both elderly males and females. However, the current literature reveals a significant gap in our knowledge regarding the sex-associated differences in gut microbiota composition among the healthy elderly population. Limited published studies on humans showing sex differences in the gut microbiome often included subjects over a broad age range, complicating interpretation for the elderly population [1]. The situation is further complicated by inconsistent and inconclusive results, with some studies reporting significant associations between sex and the gut microbiota [13,14], while others do not [11,15]. Moreover, the same microbial taxa showed opposing trends in relative abundance (increase or decrease) between studies. For example, while Hopkins et al. observed an increase in Bacteroides species in the feces of elderly subjects, Woodmansey et al. reported a decline in these species among them [16,17]. Adding to the complexity, many of these studies used 16S rRNA amplicon sequencing, which exclusively targeted bacteria [11,12,13,14]. Thus, while titles refer broadly to “gut microbiota”, the analyses are limited to bacteria, potentially overlooking sex-associated variations in other microbial domains such as archaea, fungi, and viruses. Moreover, published studies vary in the taxonomic levels at which findings are reported, limiting comparability across studies.

These limitations and inconsistencies in the current literature highlight uncertainty regarding sex-associated patterns of the gut microbiota in the healthy elderly. Therefore, it is important to directly assess and characterize potential sex-specific differences within this population. Improved knowledge of sex-specific composition and sex-associated similarities and differences in gut microbiota among healthy elderly may not only contribute to the development of effective intervention strategies for healthy aging but also aid in understanding disease susceptibility and informing the progression, prevention, and management of various diseases in this vulnerable population.

In light of these considerations, this study aimed to examine sex-associated similarities and differences in gut microbiota diversity across six taxonomic levels in a cohort of apparently healthy elderly Japanese males and females, using shotgun metagenomic sequencing. Additionally, we sought to identify sex-stratified dominant microbial profiles within this cohort.

## 2. Materials and Methods

### 2.1. Participants

This study was conducted in accordance with the Declaration of Helsinki, and the study protocol was approved by the Institutional Review Board of Yamaguchi University (Approval No. 2020-183-3). Written informed consent was obtained from all participants prior to enrollment. The data presented here are based on partial baseline analyses from a cohort study examining the effects of exercise intervention and yogurt intake in elderly individuals. Participants were apparently healthy community-dwelling elderly individuals aged 75–83 years at the time informed consent was obtained. In the present study, participants were considered apparently healthy if they were community-dwelling, able to complete the study assessments, and had no severe, acute, or unstable disease requiring active medical intervention at enrollment. Common chronic conditions that were stable and medically controlled (with or without medication), such as hypertension, diabetes, and metabolic abnormalities, were not treated as exclusion criteria, as these conditions are highly prevalent in this age group. Participants were recruited from Ajisu City, Yamaguchi Prefecture, Japan, through advertisements including posters, bulletin board notices, and local newsletters. A total of 104 volunteers initially expressed interest and were invited to participate. The inclusion criteria were: ability to stand unassisted; a Mini-Mental State Examination (MMSE) score ≥ 24; not being certified as requiring long-term nursing care level 2 or higher; absence of neurological, musculoskeletal, or connective tissue disorders; no serious medical conditions such as cancer or renal failure; and no regular use of antibiotics at the time of recruitment.

The study protocol was explained in detail to potential participants at a designated location. After informed consent was obtained, the MMSE was administered and long-term nursing care certification status was confirmed. Among the 104 volunteers, two withdrew due to procedural difficulties and two were excluded due to the need for medical treatment. The remaining 100 participants (54 males, 46 females) met all eligibility criteria and were enrolled in the study.

Following enrollment, participants underwent physical examinations and clinical and laboratory testing to confirm clinical stability, including screening for conditions suggestive of anemia or frailty. In addition, personal and medical history information was collected using a self-administered questionnaire.

### 2.2. Collection of Blood Samples and Hematological Analyses

A total of 55 mL of venous blood was collected from the cubital vein with the participant seated after an overnight fast. This sampling volume was approved by the institutional ethics committee and was required to obtain sufficient serum/plasma and whole-blood aliquots for the comprehensive hematological, biochemical, and biomarker assessments specified in the parent intervention protocol, of which the present report represents a subset of baseline data. Hematological analyses were conducted using the Sysmex XN-1000 (Sysmex, Kobe, Japan). Biochemical analyses were performed with the LABOSPECT 006 (Hitachi High-Technologies, Tokyo, Japan) using enzymatic methods. HbA1c was measured via high-speed ion-exchange liquid chromatography using the HLC-723 (Tosoh Co., Tokyo, Japan).

### 2.3. Collection of Stool Samples

Stool samples were collected using FS-0007 kits (TechnoSuruga Laboratory Co., Ltd., Shizuoka, Japan), which can preserve microbiota at room temperature for up to four weeks. Participants collected fecal samples at home and delivered them within four days. Upon receipt, the samples were stored at 4 °C, typically for an average of three days, in the cold storage facility of the Department of Public Health and Preventive Medicine, Yamaguchi University Graduate School of Medicine, until they were transported to Takara Bio Inc. (Shiga, Japan) for shotgun metagenomic analysis.

### 2.4. Metagenomic Library Preparation and Sequencing

From the stool samples, DNA was extracted using the NucleoSpin Soil extraction kit (Macherey-Nagel, Germany) following the manufacturer’s instructions. Prior to library construction, the quality and quantity of DNA samples were verified through agarose gel electrophoresis and fluorescence quantification using the Qubit 2.0 Fluorometer (Thermo Fisher Scientific, Waltham, MA, USA), respectively. DNA samples were mechanically fragmented to the desired insert size using the Covaris acoustic DNA shearing system (Covaris, Inc., Woburn, MA, USA). AMPure XP beads (Beckman Coulter Inc., Brea, CA, USA) were then employed for DNA selection based on size. After smoothing and phosphorylating the ends of the size-selected DNA, adapters were ligated, using ThruPLEX DNA-Seq Kit (Takara Bio Inc, Shiga, Japan). The DNA with ligated adapters served as a template, and PCR amplification was conducted using primers with indexes to create a sequencing library.

The quality of the generated sequencing library was assessed using the Agilent 2100 TapeStation system (Agilent, Santa Clara, CA, USA). The DNA libraries were sequenced across four lanes of an Illumina NovaSeq 6000 system (Illumina, Inc., San Diego, CA, USA) with a read length of 150 bp (paired-end). Real-time analysis was performed using NovaSeq Control Software v1.7.5 and Real Time Analysis v3.4.4 ((Illumina, Inc., San Diego, CA, USA), both executed on the NovaSeq instrument computer. To generate the sequence data, the bcl2fastq 2.20.0.422 pipeline was employed ((Illumina, Inc., San Diego, CA, USA). Per-sample shotgun metagenomic sequencing and read-processing metrics are provided in Supplementary Table S1.

### 2.5. Bioinformatic Analysis

Trimmomatic (v0.39) was utilized to trim all paired-end reads in raw FASTQ files, and low-quality reads were removed. All sequence reads were mapped to the existing human reference genome using Bowtie2 v2.2.5 (Johns Hopkins University, Baltimore, MD, USA), and host reads were eliminated. Taxonomic classification of the resulting shotgun metagenomic reads was performed using Kaiju, a protein-level fast and sensitive metagenomic classifier, against the NCBI non-redundant (nr) database. Because Kaiju classifies reads against NCBI-nr, the analysis inherently included taxa from bacteria, archaea, fungi, viruses, and eukaryotes, and no microbial domains were excluded a priori. Hits matched with NCBI Taxonomy ID were converted to taxonomic names using TaxonKit, and the relative abundance of microbial communities in each sample were calculated based on the number of reads assigned to each taxon.

### 2.6. Statistical Analysis

The continuous variables in this study were assessed for normal distribution using the Kolmogorov–Smirnov and Shapiro–Wilk tests. Subsequently, non-parametric tests were employed. In tables and figures (except those reporting microbial taxa abundance), continuous variables are presented as the median and interquartile range (IQR), while categorical variables are expressed as numbers and percentages. The comparison between male and female groups was performed utilizing the Mann–Whitney U-test for continuous variables and the Chi-square (χ^2^) test for categorical variables. Alpha diversity metrics, including the Shannon index, Simpson index, and Chao 1 index, were measured. These analyses were based on the relative abundance at each taxonomic level. The differential distribution of the overall microbial community between males and females was assessed by measuring beta diversity. Principal coordinate analysis (PCoA) of quarter root-transformed data based on the Bray–Curtis similarity matrix was conducted for this purpose. PCoA plots were generated using the first two principal coordinates (PCs), illustrating the differences between male and female groups. Additionally, group differences were examined using permutational multivariate analysis of variance (PERMANOVA) with 999 permutations. The analyses were performed using Past version 4.03 [18].

The top ten most abundant microbial taxa at each taxonomic level were identified separately for male and female groups, excluding unclassified taxa to focus on well-defined taxonomic units. For this purpose, we calculated the average percent relative abundances and presented the data as mean ± standard error (SE), consistent with common reporting formats in gut microbiota studies. However, in line with the statistical framework of this study, differences in the relative abundances of taxonomic units between sexes were assessed using non-parametric (Mann–Whitney U-test) tests.

In addition to PAST, SPSS version 22 for Windows (SPSS Inc., Chicago, IL, USA) was employed for data analysis. All statistical tests adopted in this study were two-tailed, and the significance level was considered at *p* < 0.05. To account for multiple comparisons in taxonomic differential-abundance testing, *p*-values obtained from the Mann–Whitney U-test for taxa were additionally adjusted using the Benjamini–Hochberg false discovery rate (FDR) method.

## 3. Results

Table 1 illustrates the demographic and clinical characteristics of the study population based on sex. Both males and females exhibited a similar age and BMI (Mann–Whitney U-test, *p* > 0.05). Additionally, no statistically significant differences were observed in any clinical variables between the two groups (Mann–Whitney U-test, all *p* > 0.05).

**Figure 1:**
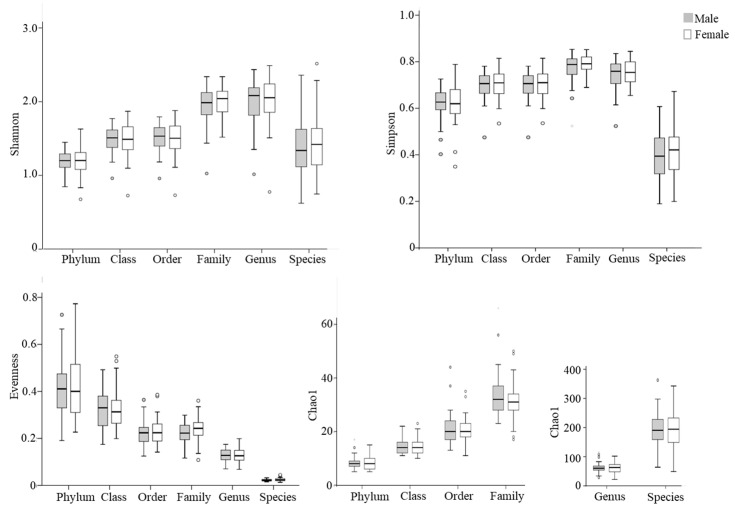
Box plots of alpha diversity indices of gut microbiota in elderly males (shaded bars; n = 54) and females (open bars; n = 46). The Shannon index, Simpson index, and evenness are presented as unitless values, while the Chao1 index is shown as the estimated number of taxa, reflecting species richness. Outliers are indicated by circles.

Next, alpha diversity of the microbial community was compared between males and females (Figure 1). No significant differences in Shannon, Simpson, or Chao 1 indexes were observed between male and female populations at different taxonomic levels (*p* > 0.05).

**Figure 2:**
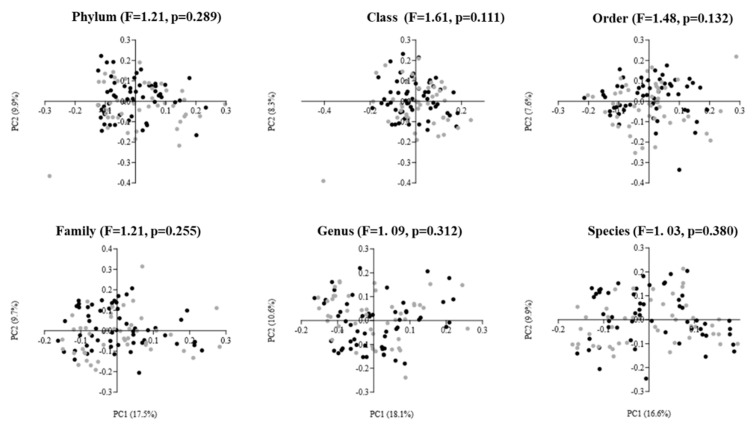
Principal coordinate analysis (PCoA) plots of gut microbiota in elderly males (black dots; n = 54) and females (gray dots; n = 46) using Bray–Curtis dissimilarity index at different taxonomic levels. The PCoA results are depicted as two-dimensional plots, with the axes indicating the percentage of data variance explained by the first two principal coordinate dimensions. The results of the PERMANOVA, comparing dissimilarity indices among groups, are displayed at the top of the plots for each corresponding taxonomic level.

The results of beta diversity are presented as two-dimensional plots using PCoAs (Figure 2). The analysis revealed that the gut microbiota of males and females did not cluster away from each other. This was confirmed by the one-way PERMANOVA test, which showed no statistically significant difference in beta diversity at any taxonomic level between elderly males and females (PERMANOVA: F = 1.03 to 1.61, *p* = 0.111 to 0.380).

Finally, we examined whether the relative abundance of the top ten most abundant taxa at each taxonomic level differed between male and female participants (Table 2). At the phylum level, in both male and female groups, the same taxa comprised the top ten list, with *Bacillota* (formerly *Firmicutes*) and *Bacteroidota* (formerly *Bacteroidetes*) as the core taxa making up the majority of the gut microbial community (>70% combined). At this level, no specific phyla were identified that differed significantly in relative abundance between males and females, except for *Bacteroidota*, the relative abundance of which was higher in males than in females (Mann–Whitney U-test, unadjusted *p* < 0.05; Table 2). At the class level, the most abundant microbial taxa were the same in both males and females, with Bacteroidia and Clostridia comprising the majority (>60% combined) of the gut microbiota. Compared with females, Bacteroidia and Betaproteobacteria showed a higher abundance in the male subjects (Mann–Whitney U-test, unadjusted *p* < 0.05; Table 2). When classified according to the order, OTU abundance revealed that Bacteroidales, Clostridiales, and Bifidobacteriales dominated the gut microbiota composition in both males and females (>70%). On the other hand, the relative abundance of Bacteroidales and Burkholderiales was higher in fecal samples of males compared to females (Mann–Whitney U-test, unadjusted *p* < 0.05; Table 2). However, after Benjamini–Hochberg FDR correction for multiple testing, none of the observed taxonomic differences in Table 2 remained statistically significant.

At the family and genus levels, nine, and at the species level, six of the top ten abundant taxa were common in both males and females (Table 2). However, the two groups of elderly subjects did not demonstrate any significant differences in any of the included taxa at these three (Family, Genus, Species) taxonomic levels.

## 4. Discussion

In humans, studying sex-related differences in gut microbiota proves challenging due to the influence of both biological sex and socio-environmental factors [11]. Therefore, in the current study, we deliberately selected a population with similar sociodemographic and clinical characteristics (as reflected in Table 1) to enhance the robustness of our findings in elucidating sex-related differences in gut microbiota.

In this study, we did not detect any differences in the alpha diversity of gut microbial communities between males and females at any taxonomic level. In a relatively large dataset of gut microbiota analyzed at the genus level using 16S rRNA gene sequencing among Japanese adults aged 70 years and older, no significant differences in α-diversity were observed between males and females [19]. Similarly, Haro et al. did not observe any differences in alpha diversity between males and females when the average age of the participants was around 60 years [20]. In contrast, a Chinese study conducted among a cohort including both young and old adults exhibited a higher Shannon index of alpha diversity for the fecal microbiota of females [14]. Similarly, in a population-based cohort study conducted among participants from the northern provinces of the Netherlands, with an age range of 18–81 years, the authors showed that females had a greater Shannon index of diversity [21]. Consistent with these findings, in a study of male and female populations aged 48.7 ± 10.2 to 53.8 ± 7.7 years, Borgo et al. observed a decreased alpha diversity at the species level in the mucosa-associated gut microbiota of male subjects [22]. Conversely, a Korean study by Shin et al. conducted among apparently healthy subjects aged 25 to 65 years and a Japanese study by Takagi et al. among healthy subjects aged 20 to 89 years did not reveal any differences between males and females in the alpha diversity of gut microbiota [11,15].

The earlier discussion of the relevant literature indicates that sex-specific differences in gut microbial alpha diversity are inconsistent and sometimes contradictory. It is also difficult to directly compare our findings on gut microbial alpha diversity with those of other studies, as the majority of published research includes both young and older subjects or centenarians, whereas our study focused solely on the elderly. Additionally, many of these studies did not clearly report the taxonomic level at which sex-related differences in gut microbial diversity were observed, or they did not conduct analyses across all taxonomic levels. However, considering both the findings from the existing literature and the results of our study, we posit that, among the elderly, sex-related differences in gut microbial alpha diversity are likely to be fairly subtle.

In our study, PCoA for beta diversity did not reveal distinct clustering patterns for elderly males and females. This aligns with findings from five separate studies that utilized PCoA showing no separation between males and females [11,12,19,22,23]. Similarly, PCoA did not demonstrate significant grouping based on sex in the colonic microbiota among healthy participants aged 7 to 52 years across five northern European countries [24]. Therefore, it seems plausible that, regardless of subjects’ age, sex does not have a significant impact on the beta diversity of gut microbiota in the elderly.

At the phylum level, we observed a higher abundance of *Bacteroidota* among elderly males compared with elderly females. Koliada et al. also studied sex differences in the phylum-level human gut microbiota composition among subjects aged 0–60+ years and reported a significant decrease in the relative abundance of *Bacteroidota* in females compared to males [25]. However, this finding contradicts those of Gao et al. (2018), Shin et al. (2019), and Haro et al. (2016), who did not identify any differences in the relative abundance of specific taxa at the phylum level between the two sex groups [11,14,20].

In our study, *Bacteroidia* and *Betaproteobacteria*, at the class level, and *Bacteroidales* and *Burkholderiales*, at the order level, showed higher abundances in the fecal samples of elderly males. However, these between-sex differences were nominal (unadjusted *p* < 0.05) and did not remain statistically significant after Benjamini–Hochberg false discovery rate correction, suggesting that any sex-associated taxonomic shifts in this cohort are subtle. On the other hand, Gao et al. (2018) did not observe any significant taxonomic differences at the class or order levels between males and females [14].

In our study, at the Family and Genus taxonomic levels, the gut microbiota composition with respect to the most abundant microbial taxa was found to be similar between elderly males and females. In the available literature, a number of published studies have reported the sex-specific composition and relevant differences in gut microbiota, especially at the genus taxonomic level, although these findings are often inconsistent. For example, Haro et al. observed a higher presence of the genera *Veillonella* and *Methanobrevibacter* in fecal samples from males compared to females, while the abundance of the genus *Bilophila* was higher in females [20]. Santos-Marcos et al. found higher levels of the genera *Ruminococcus* and *Bilophila* in healthy females, and higher levels of the genera *Clostridium* and *SMB53* in males than in their opposite-sex groups [26]. Gao et al. and Takagi et al. also observed a higher abundance of the genus *Ruminococcus* in fecal samples from females compared to males [14,15]. Moreover, Takagi et al. observed elevated numbers of the genera *Prevotella, Megamonas*, *Megasphaera*, and *Fusobacterium* in males, while *Bifidobacterium* and *Akkermansia* were detected at higher levels in females [14,15]. In a study of a population aged 24 to 100 years, Mueller et al. observed gender effects at the genus level for the *Bacteroides-Prevotella* group, with higher levels in males [27]. In contrast, Singh and Manning, in their study among healthy individuals including elderly people, observed that the genus *Paraprevotella* was differentially abundant in the intestinal microbial community of males, whereas the genera *Dialister*, *Lactobacillus*, unclassified *Lactobacillaceae*, unclassified *Comamonadaceae*, and *Comamonas* were differentially abundant in females [12].

At the species level, six taxa were found to be common in both elderly males and females in our study, and no significant gender differences were observed for any of the top 10 taxa. At this taxonomic level, compared with females, a higher abundance of *Bacteroides plebeius* (in subjects with BMI > 33 kg/m^2^) and *Coprococcus catus* (in subjects with BMI < 30 kg/m^2^) was found among males in the study by Haro et al. [20]. Meanwhile, *Bacteroides thetaiotaomicron*, *Eubacterium eligens*, *Klebsiella pneumoniae*, *Sutterella stercoricanis*, *Ruminococcus obeum* and *Ruminococcus bromii* showed higher abundances among males in the study by Li et al. [28]. In contrast, after adjusting for different confounding factors, Sinha et al. revealed that *Akkermansia muciniphila* was associated with sex, with females having a higher abundance of this species [21].

All the aforementioned findings demonstrate the existence of discrepancies in the current literature regarding the composition and abundance of dominant gut microbiota in male versus female subjects at various taxonomic levels. These variations in the reported findings may, in part, stem from the inclusion of subjects across diverse races, ages, and other demographic and socio-environmental factors, as well as differences in health status and diet [29]. However, we observed a similarity in gut microbial diversity among elderly males and females, with only small differences in the gut microbial composition at three different taxonomic levels. The current study design precludes elucidating the mechanisms responsible for the disparities observed between studies regarding gut microbiota composition and diversity between males and females. It has been mentioned that sex-specific steroid hormones, such as estradiol in females and testosterone in males, could play pivotal roles in modulating gut microbial composition and differentiation between the sexes [11,30,31]. As aging progresses, there is a gradual but robust decline in testosterone production in males and almost complete loss of estrogen at menopause in females [32,33,34,35]. Therefore, it is possible that the age-related decline in circulating levels of primary sex steroid hormones in both males and females likely contributes to the decrease or disappearance of gut microbial sex differences among the elderly.

In reporting our findings, we presented the relative abundance data as mean ± SE. This approach aligns with common reporting practices in microbiome research, where mean values are widely used to summarize relative abundance data to enhance clarity and facilitate comparison across studies [17,20,22,26]. However, as noted by Gloor et al. (2017) and Weiss et al. (2017), the compositional nature of microbiome data requires cautious interpretation of such summary statistics [36,37]. Also, interpretations of our findings should also be considered in light of several potential limitations. Firstly, detailed information on diet and lifestyle was not available for this analysis. Such factors are known to influence gut microbiota composition and may also differ between males and females. Therefore, residual confounding cannot be excluded and could have attenuated or obscured modest sex-associated differences. Nevertheless, because participants were community-dwelling elderly recruited from the same geographic area and shared broadly similar socio-demographic characteristics, large between-sex differences in these exposures were likely reduced in this cohort. Future studies incorporating standardized dietary/lifestyle assessments will be important to better distinguish biological sex effects from behavioral and environmental influences. Secondly, the cross-sectional design of our study precludes any causal inference between sex and the gut microbiome. Another potential limitation is the relatively small sample size. Although the present sample size (n = 100) is acceptable for shotgun metagenomic profiling in an elderly cohort, the study may have been underpowered to detect subtle sex-associated differences at lower taxonomic levels (e.g., genus and species). Larger cohorts or multi-center studies will be necessary to validate these findings and to determine whether more granular taxonomic or functional sex-related differences emerge in advanced age. Nevertheless, we believe that our study adds useful evidence on sex-associated patterns of the gut microbiota among the elderly. Lastly, since this study was conducted exclusively among a Japanese elderly population, the generalizability of the observed findings may be limited.

## 5. Conclusions

This study revealed that gut microbial diversity and overall community structure are largely similar between apparently healthy elderly Japanese males and females, with only modest sex-associated differences observed in the relative abundance of selected taxa at higher taxonomic levels. These results suggest that, in advanced age, biological sex has a limited influence on gut microbiota composition at the population level. Accordingly, our findings provide population-level reference data for the gut microbiota of older adults and indicate that sex alone may not be a primary stratification factor when considering microbiota-modulating strategies aimed at promoting healthy aging. Future longitudinal and interventional studies are warranted to evaluate whether responses to microbiota-targeted interventions differ meaningfully by sex in older populations and to identify host or environmental factors that may better inform stratification and personalization.

## Figures and Tables

**Table 1:** Demographic and clinical characteristics of the study subjects. Values are expressed as median and IQR.

	Male, n = 54		Female, n = 46		*p* *
Variables	Median	IQR	Median	IQR	
Age (Years)	77.5	3.0	77.0	2.0	0.812
BMI (kg/m^2^)	23.5	4.4	22.6	3.3	0.407
FPG (mg/dL)	101.0	16.0	97.5	17.3	0.653
AST (U/L)	25.5	7.0	23.5	9.0	0.136
ALT (U/L)	18.0	10.3	18.0	10.5	0.750
GGT (U/L)	25.5	21.0	19.5	14.0	0.114
HDLC (mg/dL)	66.4	31.9	64.4	29.3	0.917
LDLC (mg/dL)	127.5	33.0	118.0	39.5	0.675
TG (mg/dL)	89.5	53.8	102.0	54.3	0.356
UA (mg/dL)	5.6	1.1	5.5	2.4	0.263

BMI, body mass index; ALT, Alanine aminotransferase; AST, aspartate aminotransferase; FPG, fasting plasma glucose; GGT, gamma-glutamyl transpeptidase; HDLC, high-density lipoprotein cholesterol; LDLC, low-density lipoprotein cholesterol; UA, uric acid; TG, triglyceride. * Two-tailed *p*-values were obtained using the Mann–Whitney U-test for two independent samples.

**Table 2:** (**a**) Percent relative abundances (mean ± SE) of the top ten most abundant taxa (ranked by overall abundance) at phylum, class and order taxonomic levels in elderly male and female participants. (**b**) Percent relative abundances (mean ± SE) of the top ten most abundant taxa (ranked by overall abundance) at family, genus and species taxonomic levels in elderly male and female participants.

(**a**)				
	**Male**		**Female**	
**Taxa**	**Gut Microbiota**	**Mean ± SE**	**Gut Microbiota**	**Mean ± SE**
Phylum	*Bacillota*	44.01 ± 1.76	*Bacillota*	46.48 ± 2.05
	*Bacteroidota* *	36.07 ± 1.67	*Bacteroidota*	30.36 ± 1.75
	*Actinomycetota*	7.08 ± 0.99	*Actinomycetota*	10.03 ± 1.98
	*Pseudomonadota*	3.46 ± 0.53	*Pseudomonadota*	2.75 ± 0.48
	*Verrucomicrobiota*	0.51 ± 0.16	*Verrucomicrobiota*	1.48 ± 0.48
	*Fusobacteriota*	0.12 ± 0.08	*Euryarchaeota*	0.12 ± 0.05
	*Chlamydiota*	0.05 ± 0.01	*Synergistota*	0.10 ± 0.05
	*Euryarchaeota*	0.03 ± 0.01	*Fusobacteriota*	0.09 ± 0.05
	*Synergistota*	0.02 ± 0.01	*Lentisphaerota*	0.04 ± 0.02
	*Lentisphaerota*	0.01 ± 0.00	*Chlamydiota*	0.04 ± 0.01
Class	*Bacteroidia* *	35.77 ± 1.66	*Clostridia*	35.86 ± 1.87
	*Clostridia*	33.27 ± 1.47	*Bacteroidia*	29.98 ± 1.73
	*Actinobacteria*	5.32 ± 0.89	*Actinobacteria*	8.40 ± 1.96
	*Bacilli*	3.09 ± 0.53	*Bacilli*	2.89 ± 0.45
	*Gammaproteobacteria*	2.35 ± 0.50	*Gammaproteobacteria*	1.79 ± 0.46
	*Negativicutes*	1.74 ± 0.22	*Coriobacteriia*	1.59 ± 0.2
	*Coriobacteriia*	1.73 ± 0.17	*Verrucomicrobiae*	1.47 ± 0.48
	*Betaproteobacteria* *	0.51 ± 0.05	*Negativicutes*	1.27 ± 0.17
	*Verrucomicrobiae*	0.51 ± 0.16	*Betaproteobacteria*	0.41 ± 0.08
	*Erysipelotrichia*	0.40 ± 0.06	*Erysipelotrichia*	0.4 ± 0.06
Order	*Bacteroidales* *	35.74 ± 1.66	*Clostridiales*	35.47 ± 1.85
	*Clostridiales*	33.05 ± 1.47	*Bacteroidales*	29.96 ± 1.73
	*Bifidobacteriales*	5.14 ± 0.89	*Bifidobacteriales*	8.22 ± 1.96
	*Lactobacillales*	2.89 ± 0.53	*Lactobacillales*	2.75 ± 0.45
	*Enterobacterales*	2.00 ± 0.46	*Enterobacterales*	1.55 ± 0.42
	*Coriobacteriales*	1.53 ± 0.16	*Verrucomicrobiales*	1.47 ± 0.48
	*Veillonellales*	0.84 ± 0.16	*Coriobacteriales*	1.31 ± 0.20
	*Acidaminococcales*	0.51 ± 0.07	*Veillonellales*	0.71 ± 0.15
	*Burkholderiales* *	0.51 ± 0.05	*Acidaminococcales*	0.45 ± 0.08
	*Verrucomicrobiales*	0.51 ± 0.16	*Burkholderiales*	0.41 ± 0.08
(**b**)				
	**Male**		**Female**	
**Taxa**	**Gut Microbiota**	**Mean ± SE**	**Gut Microbiota**	**Mean ± SE**
Family	*Bacteroidaceae*	17.13 ± 1.75	*Bacteroidaceae*	15.59 ± 1.44
	*Prevotellaceae*	9.91 ± 1.82	*Ruminococcaceae*	10.04 ± 0.83
	*Ruminococcaceae*	8.92 ± 0.84	*Bifidobacteriaceae*	7.91 ± 1.91
	*Lachnospiraceae*	6.43 ± 0.43	*Lachnospiraceae*	6.78 ± 0.53
	*Bifidobacteriaceae*	4.91 ± 0.83	*Prevotellaceae*	5.63 ± 1.63
	*Rikenellaceae*	3.37 ± 0.93	*Rikenellaceae*	3.29 ± 0.62
	*Streptococcaceae*	2.27 ± 0.51	*Eubacteriaceae*	2.08 ± 0.27
	*Clostridiaceae*	1.9 ± 0.18	*Clostridiaceae*	1.93 ± 0.23
	*Eubacteriaceae*	1.82 ± 0.17	*Streptococcaceae*	1.73 ± 0.34
	*Coriobacteriaceae*	1.49 ± 0.16	*Akkermansiaceae*	1.47 ± 0.48
Genus	*Bacteroides*	17.04 ± 1.75	*Bacteroides*	15.51 ± 1.43
	*Prevotella*	9.62 ± 1.80	*Bifidobacterium*	7.85 ± 1.90
	*Bifidobacterium*	4.87 ± 0.83	*Prevotella*	5.26 ± 1.61
	*Faecalibacterium*	3.41 ± 0.36	*Ruminococcus*	3.74 ± 0.58
	*Ruminococcus*	3.30 ± 0.49	*Faecalibacterium*	3.41 ± 0.37
	*Alistipes*	3.10 ± 0.90	*Alistipes*	3.03 ± 0.58
	*Streptococcus*	2.22 ± 0.49	*Eubacterium*	2.01 ± 0.27
	*Eubacterium*	1.74 ± 0.17	*Streptococcus*	1.63 ± 0.34
	*Clostridium*	1.60 ± 0.17	*Clostridium*	1.63 ± 0.22
	*Collinsella*	1.47 ± 0.15	*Akkermansia*	1.47 ± 0.48
Species	*Prevotella copri*	3.44 ± 0.68	*Prevotella copri*	1.82 ± 0.6
	*Faecalibacterium prausnitzii*	1.44 ± 0.17	*Faecalibacterium prausnitzii*	1.52 ± 0.20
	*Bifidobacterium adolescentis*	0.64 ± 0.14	*Bifidobacterium adolescentis*	1.2 ± 0.35
	*Bacteroides plebeius*	0.48 ± 0.11	*Bifidobacterium longum*	0.66 ± 0.21
	*Bifidobacterium longum*	0.40 ± 0.10	*Bacillota bacterium CAG:124*	0.52 ± 0.20
	*Bacteroides stercoris*	0.38 ± 0.09	*Akkermansia muciniphila*	0.44 ± 0.14
	*Bacteroides vulgatus*	0.38 ± 0.06	*Bacteroides plebeius*	0.43 ± 0.16
	*Ruminococcus* sp. *CAG:254*	0.29 ± 0.10	*Subdoligranulum* sp. *APC924/74*	0.39 ± 0.09
	*Subdoligranulum* sp. *APC924/74*	0.28 ± 0.05	*Gemmiger formicilis*	0.37 ± 0.07
	*Eubacterium* sp. *CAG:180*	0.27 ± 0.06	*Bacteroides fragilis*	0.35 ± 0.11

* Indicates unadjusted *p* < 0.05 (Mann–Whitney U-test).

## Data Availability

The data generated in this study are not currently publicly available due to data-governance agreements with collaborating partners. De-identified, host-filtered sequencing data and/or other datasets supporting the findings are available from the corresponding author upon reasonable request, subject to approval by the relevant parties and, where applicable, execution of a data-sharing agreement.

## Supplementary Materials

The following supporting information can be downloaded at: https://www.mdpi.com/article/10.3390/life16020297/s1, Table S1: Per-sample shotgun metagenomic sequencing and read-processing metrics.
